# Simplified Method for Predicting a Functional Class of Proteins in Transcription Factor Complexes

**DOI:** 10.1371/journal.pone.0068857

**Published:** 2013-07-12

**Authors:** Marek J. Piatek, Michael C. Schramm, Dharani D. Burra, Abdulaziz binShbreen, Boris R. Jankovic, Rajesh Chowdhary, John A.C. Archer, Vladimir B. Bajic

**Affiliations:** 1 King Abdullah University of Science and Technology (KAUST), Computer, Electrical and Mathematical Sciences and Engineering Division, Computational Bioscience Research Center, Thuwal, Kingdom of Saudi Arabia; 2 Biomedical Informatics Research Center, MCRF, Marshfield Clinic, Marshfield, Wisconsin, United States of America; Kyushu Institute of Technology, Japan

## Abstract

**Background:**

Initiation of transcription is essential for most of the cellular responses to environmental conditions and for cell and tissue specificity. This process is regulated through numerous proteins, their ligands and mutual interactions, as well as interactions with DNA. The key such regulatory proteins are transcription factors (TFs) and transcription co-factors (TcoFs). TcoFs are important since they modulate the transcription initiation process through interaction with TFs. In eukaryotes, transcription requires that TFs form different protein complexes with various nuclear proteins. To better understand transcription regulation, it is important to know the functional class of proteins interacting with TFs during transcription initiation. Such information is not fully available, since not all proteins that act as TFs or TcoFs are yet annotated as such, due to generally partial functional annotation of proteins. In this study we have developed a method to predict, using only sequence composition of the interacting proteins, the functional class of human TF binding partners to be (i) TF, (ii) TcoF, or (iii) other nuclear protein. This allows for complementing the annotation of the currently known pool of nuclear proteins. Since only the knowledge of protein sequences is required in addition to protein interaction, the method should be easily applicable to many species.

**Results:**

Based on experimentally validated interactions between human TFs with different TFs, TcoFs and other nuclear proteins, our two classification systems (implemented as a web-based application) achieve high accuracies in distinguishing TFs and TcoFs from other nuclear proteins, and TFs from TcoFs respectively.

**Conclusion:**

As demonstrated, given the fact that two proteins are capable of forming direct physical interactions and using only information about their sequence composition, we have developed a completely new method for predicting a functional class of TF interacting protein partners with high precision and accuracy.

## Introduction

The central dogma of biology revolves around three major processes: DNA replication, transcription, and translation [1]. Initiation of transcription is in the core of most of the cellular responses to environmental conditions [2]. It also determines the cell and tissue specificity [3]. The focus of this study is functional annotation of nuclear proteins within transcription factor (TF) complexes. Transcription initiation is a highly dynamic and regulated process [4]. It involves interactions of different transcription-associated proteins, ligands and TFs forming protein complexes that cooperatively act to create the environment allowing RNA polymerase to initiate transcription. Nuclear proteins from different functional classes interact with TFs during the transcription initiation [5]. Among these, our focus is on TFs, transcription co-factors (TcoFs), and other nuclear proteins that cannot be classified being either TFs or TcoFs. We follow the definition of TcoF by [6] where it is defined as a protein that binds to TF, modulates transcription initiation through such a complex and itself does not bind DNA.

Studying protein interactions has been a field of major interest in systems biology and bioinformatics over the past few years [7]–[9]. These studies are based on a combination of laboratory experiments with proven interactions [10] and studies based on these proven interactions in order to investigate potentially novel protein-protein interactions (PPIs), so as to build predictive models [11]. Studies such as [12] revealed some potentially general rules that govern interactions between two proteins. Such rules are termed PPI Indicators. PPI Indicators are detected at various levels including the genomic context through evolution, the level of physico-chemical properties of amino acids, and the structural level [13]. Thus, it can be observed that the PPIs are based on physico-chemical parameters of amino acids present in the interacting proteins and sequence/structural specific information that govern their interactions. Although a number of physico-chemical parameters have been used to study PPIs, not all of them exhibited high resolution in resolving and identifying PPIs [14]. The same is observed with the sequence/structure based identifiers [14]. PPI studies have focused on the proteome interactome at the cellular/tissue levels, while very few studies have investigated the interactions between specific protein subsets, for example, proteins involved in a specific process [15], [16]. It was hypothesized [17] that there could be an over-representation of certain interaction rules within subsets of specific proteins from the global PPI map owing to the difference in the activities performed by these protein subsets. However, to the best of our knowledge, little attention has been devoted to interactions within specific classes of proteins.

The transcription process is one of the most challenging regarding studies of PPIs due to its highly dynamic nature that involves multiple nuclear proteins. Extensive research has been conducted on TF and DNA interactions, both computationally and experimentally [18]. Work has also been carried out in identifying interactions between two TFs [16], [19]. However, the problem we study here is not prediction of the binding of two proteins. Rather we study the functional identity/characteristics of the TF binding proteins. This can help us to better annotate proteins involved in transcription regulation, which ultimately helps in analysis and understanding of transcription regulation process. To the best of our knowledge, no similar study has been published to date. For example, our system suggests that Exophilin-5 (EXPH5) and Cleavage and polyadenylation specificity factor subunit 6 (CPSF6) could be classified as TcoFs, while suppression of tumorigenicity 5 (ST5) and Proteasomal ubiquitin receptor ADRM1 could additionally be classified as TF.

The aim of this research is to develop a simplified method to predict the functional class of a protein that binds a TF. The functional classes we consider here are: (i) TF, (ii) TcoF, or (iii) other nuclear protein. For this functional class prediction we use only the amino acid sequence properties of the interacting proteins. Our bioinformatics-based approach analyses three sets of binding protein pairs: TF-TcoF, TF-TF, and TF-other (protein). We have been successful in using amino acid-based physico-chemical information contained in the binding protein pairs to build models capable of identifying and differentiating the above-mentioned categories of interacting proteins. Our classification systems achieved high accuracies [20] in distinguishing TFs and TcoFs from other nuclear proteins, and TFs from TcoFs respectively. In addition, we developed a web application that performs these predictions, which is publically accessible at http://www.cbrc.kaust.edu.sa/tftcofc. Our method can help in complementing annotation of the currently known pool of nuclear proteins. Since only the knowledge of protein sequences is required in addition to protein interaction, the method, in principle, should be easily applicable to many species.

## Results

Experimentally validated binding between TF and TcoF, as well as between TF and TF, and TF and other nuclear proteins from TcoF-DB [6] were used to develop our models (more details in Supplementary Table 2 and 3 in Materials S1). All unique instances of interactions from TcoF-DB were classified into three cases:

- TF-TcoF: Here, the first of the binding proteins is a TF and the second is a TcoF. A total of 2401 instances were identified.
- TF-TF: Here, the first of the binding proteins is a TF and the second is a TF. A total of 1156 instances were identified.
- TF-other: Here, the first of the binding proteins is TF and the second is another nuclear protein (not TF, not TcoF). A total of 3437 instances were identified.

The amino acid sequences for all binding proteins were extracted from the Universal Protein Resource database [21] (http://www.uniprot.org/).

The Amino Acid Index database [22] (http://www.genome.jp/aaindex/) consists of 544 physico-chemical characteristics for each amino acid. Based on the previous PPI studies [11], [12] we used two sets of these characteristics for our experiments. The one with 171 characteristics (see Supplementary Table 1 in Materials S1) selected based on our assessment of relevance of these characteristics for protein-protein interactions, and the other that used all 544 characteristics. A performance comparison of different classifiers (Random Forest, J48, Bayesian Network, Naïve Bayes classifier, RBF Network, LibSVM) with these two feature sets is shown in Supplementary Table 6, 7 and 8 in Materials S1).

The values of all the amino acid properties obtained for the interacting protein pairs were used to build a feature vector for each instance of binding proteins. The feature vectors consisting of 171 features for all the instances were used to develop efficient predictive models for our problem. We performed various experiments (Supplementary Tables 5–11 in Materials S1) with the WEKA tool [23] to determine the most appropriate model for recognizing the functional class of TF binding proteins for the cases as described above.

We have developed two models, Model 1 and Model 2, to recognize the nature of the binding partner of TFs. Model 1 aims at distinguishing if the binding partner of TF is “TF or TcoF” (TF/TcoF), or if it is “other” nuclear protein. Due to the fact that we have to ensure that Model 1 produces the same prediction score if the order of the bound TFs is TFa-TFb or TFb-TFa, Model 1 was composed of two sub-models (M1.1 and M1.2) to cater for this situation. M1.1 was trained using TFa-TFb ordering in the cases when the binding partner of TF was TF, while M1.2 was trained using the reverse ordering for the same cases.

Since Model 1 separates TF binding partners to either “TF/TcoF” or “other” nuclear proteins, the second model, Model 2, attempts to distinguish if the TF binding partner is TF or TcoF. For the same reasons as for Model 1, Model 2 was composed of two sub-models (M2.1 and M2.2). M2.1 was trained using TFa-TFb ordering in the cases when the binding partner of TF was TF, while M2.2 was trained using the reverse ordering for the same cases (Supplementary Table 4 in Materials S1).

The most successful models were based on Random Forest classifiers. The accuracies of both Random Forest models based on 10-fold cross-validation were quite similar (M1.1∶92.1%, M1.2∶92.0%, M2.1∶95.8%, M2.2∶96.3%).

**Figure 1:**
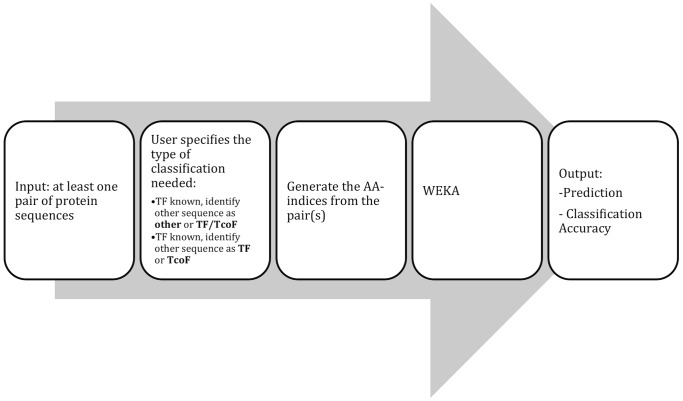
Workflow of Analysis Pipeline. Given a set of binding proteins and depending on the model type, the analysis pipeline predicts the functional identity of one of a TF binding partner. After a pair of binding proteins is given as the input, the pipeline calculates numerous amino acid physico-chemical properties for the pair and generates a matrix of feature vectors. This matrix of feature vectors is submitted to the Random Forest classifier that returns the predicted functional class assignment and the associated confidence score. The latter is a measure of how strongly the average feature-class probabilities of all decision trees pointed to each class based on the information gain of those decision trees.

We performed a number of additional experiments to assess the methodology and model stability. We randomly selected 10% of the total data for independent testing maintaining the proportion of the positive and negative cases. We then trained our models on the remaining 90% of the data and evaluated them on the extracted independent 10%. Based on these test results, our system suggests that Exophilin-5 (EXPH5), Cleavage and polyadenylation specificity factor subunit 6 (CPSF6) could be classified as TcoFs, while suppression of tumorigenicity 5 (ST5) and Proteasomal ubiquitin receptor ADRM1 could be classified as TFs. In Supplementary Tables 9 and 10 in Materials S1 we provide additional accuracy results based on a/3-fold cross-validation, b/5-fold cross-validation, c/when the 2/3 of the data is used for training and 1/3 for testing, as well as d/when 90% of the data is used for training and 10% for testing. We chose Random Forrest models (as the best and most consistently performing ones) to implement in a web-based analysis system (workflow presented in Figure 1) available at http://www.cbrc.kaust.edu.sa/tftcofc. This system is capable of predicting the functional class of the TF binding protein when the system is provided with information on the binding protein pair, where one of proteins is a known TF. The interface allows the user to input pair(s) of binding proteins in the form of their UniProt identifiers [21] and as result the system returns the prediction results for each protein pair.

## Discussion

In this study we developed two recognition models that are able to distinguish with high accuracy functional classes of proteins binding to known TFs based on interaction of the types TF-TcoF, TF-TF, and TF-“other nuclear protein”. These models explicitly assign a functional class to the TF binding protein in an evaluated protein interaction pair for cases when one of the interacting partners is a known TF. We also implemented these predictive models as a publicly accessible web application. To the best of our knowledge there is no publicly available tool that performs similar task. It is important to note that our system predicts the functional classes of proteins based on a simplified method that uses only amino acid sequence features of the binding proteins. Our attempts to use protein domains for this problem resulted in much inferior accuracy (Supplementary Table 11 in Materials S1). This was a surprise to us, as we expected that using information on protein domains should improve the accuracy of determination of the protein classes considered. One of the reasons could be that it was not possible to associate sufficient number of relevant protein domains with all proteins from the interacting pairs we considered. However, even with the simplified representation of the protein sequences we did achieve very high accuracy, which suggests that in this type of problems the averaging of amino acid properties across the whole protein sequence, as we used in this study, may be sufficient. Similar ideas were utilized in [16].

Model 1 was able to distinguish other nuclear proteins from TF/TcoF class with specificity [20] of 93.5% for M1.1 and 93.3% for M1.2. Similar results (specificity 92.7% for M2.1 and 94.1% for M2.2) were achieved with Model 2 for the distinction between TFs and TcoFs. Regarding sensitivity [20], Model 1.1 and Model 1.2 achieve equal 90.8%, while Model 2.1 and Model 2.2 achieve 97.3%. We additionally checked the robustness of our models by using 3-fold and 5-fold cross-validations, as well as for the case when 2/3 of data was used for training and 1/3 for testing. Based on the results presented in Supplementary Table 5 in Materials S1, we conclude that our models are indeed robust.

It is important to mention that the reported specificities, sensitivities and accuracies were obtained from models built on experimentally validated data regarding protein binding, and curated functional class annotation of TFs and TcoFs. The results suggest that the limited number of 171 features we used to construct the models is sufficient for relatively high accuracy prediction of the functional class of a TF binding partner. If we did use all 544 amino acid properties from AAindex database, the accuracy remained at the level very similar to when we used 171 features as shown in Supplementary Table 8 in Materials S1. That demonstrates that the 32% of all features that we used in our reduced feature set, does contain a significant fraction of the relevant amino acid properties for this particular problem, while many of the less relevant features were eliminated.

Although the classification problem we dealt with could be formulated as a multiclass problem, we found that it is conveniently and efficiently solved as two 2-class problems. The use of multiclass models from the WEKA package did not improve classification accuracy, so we retained our original solution.

Finally, in one of the tests performed, we identified four TF interacting proteins for which our system suggests potentially additional annotation. For example, CPSF6 is known to play a role in cleavage and polyadenylation factor complex by recruiting other processing factors (see EntrezGene ID: 11052), while ADRM1 (see Entrez Gene ID: 11047) suggests that it is involved in transcription elongation process from RNA polymerase II promoters and that it is functional in the nucleus. The GO annotation for ST5 (see Entrez Gene ID: 6764) suggests that it may alter cell morphology and cell growth.

Moreover, protein function predictor tool, FuncBase Gene Function Prediction Viewer [24], provide the following putative annotation, among many, that directly or indirectly support our own function predictions for the above four proteins. These are as follows:

1. CPSF6 (Entrez Gene ID 11052):
  - FuncBase, (GO:0003712) transcription cofactor activity
  - FuncBase, (GO:0003713) transcription coactivator activity
2. EXPH5 (Entrez Gene ID 23086):
  - FuncBase, (GO:0045941, GO: 0045892) regulation of transcription
  - FuncBase, (GO:0003712, GO:0003713) transcription coactivator activity
3. ST5 (Entrez Gene ID 6764):
  - FuncBase, (GO:0016563) transcription activator activity
4. ADRM1 (Entrez Gene ID 11047):
  - FuncBase, (GO: 0003702) RNA polymerase II transcription factor activity

While the existing information about these four proteins does not contradict our hypotheses, the validation to confirm or reject them can only be done experimentally, which is beyond the scope of our bioinformatics study.

## Materials and Methods

### Retrieving Protein Sequences and Interacting Domains

UniProt identification numbers from TcoF-DB (http://www.cbrc.kaust.edu.sa/tcof/) were used to download protein sequences from UniProt database (http://www.uniprot.org/) [21].

### Data Processing

For each protein, 544 different physico-chemical properties were determined using amino acid indices [22] available at (http://www.genome.jp/aaindex/). For each of the binding partner, a feature vector was built consisting of 544 features. In a given protein sequence, the numerical value for each of the features is determined as the average value of that feature across all amino acids in that protein sequence. The list of these 544 features can be found at [21]. We also manually selected a set of 171 features we considered relevant for PPI (Supplementary Table 1 in Materials S1). These two sets are used in our study. We are aware that the manual selection of the reduced feature set is somewhat subjective and not optimal. However, we attempted to eliminate the features that might not play important role in protein binding and retain the ones that are most related in the functional way to our case. On the other hand, to the best of our knowledge, there is no published set of relevant features for the problem we studied, so we hypothesised that some of the features that could be suitable for PPI could also be relevant for our problem.

### Model Building

The WEKA tool [23] was used to build the predictive models and perform classification and validation steps. The WEKA generated models are implemented in the web system based on the use of 171 selected features. The description of exact parameters used to build models with various classifiers together with the achieved accuracy, as well as the definitions of performance measures can be found in the supplementary material.

### Web Application

A web application was developed to perform the prediction of the functional class of one of the binding proteins as discussed above. For every input pair of interacting proteins a feature vector of 171 selected amino acid features is computed, submitted to a prebuilt classification model (see Supplementary Table 1 in Materials S1) and prediction is made. Each prediction is based on the average prediction score of the two models. In case of choosing Model 1, the results of confidence score predictions’ from Model 1.1 and Model 1.2 is averaged and reported. The same strategy has been implemented for Model 2. The predicted functional class is returned to the user.

## Supporting Information

Materials S1 **Supplementary Table 1.** Listing of used 171 amino acid indices. **Supplementary Table 2.** Description of the dataset used in this study. **Supplementary Table 3.** Summary of classes used to build models. **Supplementary Table 4.** Definitions of models. **Supplementary Table 5.** Performance of various classifiers using 171 features. **Supplementary Table 6.** Performance results of various classifiers used on 544 features. **Supplementary Table 7.** Performance results of various classifiers used on 171 features. **Supplementary Table 8.** Comparison of accuracies across various classifiers using 171 and 544 features. **Supplementary Table 9.** Effect of cross validation levels on various classifiers during model building stage. **Supplementary Table 10.** Effect of percentage split levels on various classifiers during model building stage. **Supplementary Table 11.** Comparative results between domains, AAI and AAI+domains with 10-fold cross-validation.
(XLS)

## References

[1] Crick F (1970) Central dogma of molecular biology. Nature 227: 561–563. 4913914 10.1038/227561a0

[2] Shamovsky I, Nudler E (2008) New insights into the mechanism of heat shock response activation. Cellular and Molecular Life Sciences 65: 855–861. 18239856 10.1007/s00018-008-7458-y PMC11131843

[3] Lemons D, McGinnis W (2006) Genomic evolution of Hox gene clusters. Science 313: 1918–1922. 17008523 10.1126/science.1132040

[4] Hager GL, McNally JG, Misteli T (2009) Transcription dynamics. Mol Cell 35: 741–753. 19782025 10.1016/j.molcel.2009.09.005 PMC6326382

[5] Goodrich JA, Tjian R (2010) Unexpected roles for core promoter recognition factors in cell-type-specific transcription and gene regulation. Nat Rev Genet 11: 549–558. 20628347 10.1038/nrg2847 PMC2965628

[6] Schaefer U, Schmeier S, Bajic VB (2011) TcoF-DB: dragon database for human transcription co-factors and transcription factor interacting proteins. Nucleic Acids Res 39: D106–110. 20965969 10.1093/nar/gkq945 PMC3013796

[7] Skrabanek L, Saini HK, Bader GD, Enright AJ (2008) Computational prediction of protein-protein interactions. Mol Biotechnol 38: 1–17. 18095187 10.1007/s12033-007-0069-2

[8] Szklarczyk D, Franceschini A, Kuhn M, Simonovic M, Roth A, et al (2011) The STRING database in 2011: functional interaction networks of proteins, globally integrated and scored. Nucleic Acids Res 39: D561–568. 21045058 10.1093/nar/gkq973 PMC3013807

[9] Blow N (2009) Systems biology: Untangling the protein web. Nature 460: 415–418. 19606149 10.1038/460415a

[10] Ewing RM, Chu P, Elisma F, Li H, Taylor P, et al (2007) Large-scale mapping of human protein-protein interactions by mass spectrometry. Mol Syst Biol 3: 89. 17353931 10.1038/msb4100134 PMC1847948

[11] Shoemaker BA, Panchenko AR (2007) Deciphering protein-protein interactions. Part II. Computational methods to predict protein and domain interaction partners. PLoS Comput Biol 3: e43. 17465672 10.1371/journal.pcbi.0030043 PMC1857810

[12] Keskin O, Gursoy A, Ma B, Nussinov R (2008) Principles of protein-protein interactions: what are the preferred ways for proteins to interact? Chem Rev 108: 1225–1244. 18355092 10.1021/cr040409x

[13] Zhou HX, Qin S (2007) Interaction-site prediction for protein complexes: a critical assessment. Bioinformatics 23: 2203–2209. 17586545 10.1093/bioinformatics/btm323

[14] Ezkurdia I, Bartoli L, Fariselli P, Casadio R, Valencia A, et al (2009) Progress and challenges in predicting protein-protein interaction sites. Brief Bioinform 10: 233–246. 19346321 10.1093/bib/bbp021

[15] De Las Rivas J, Fontanillo C (2010) Protein-protein interactions essentials: key concepts to building and analyzing interactome networks. PLoS Comput Biol 6: e1000807. 20589078 10.1371/journal.pcbi.1000807 PMC2891586

[16] Schmeier S, Jankovic B, Bajic VB (2011) Simplified method to predict mutual interactions of human transcription factors based on their primary structure. PLoS One 6: e21887. 21750739 10.1371/journal.pone.0021887 PMC3130058

[17] Rausell A, Juan D, Pazos F, Valencia A (2010) Protein interactions and ligand binding: from protein subfamilies to functional specificity. Proc Natl Acad Sci U S A 107: 1995–2000. 20133844 10.1073/pnas.0908044107 PMC2808218

[18] Stormo GD, Zhao Y (2010) Determining the specificity of protein-DNA interactions. Nat Rev Genet 11: 751–760. 20877328 10.1038/nrg2845

[19] Ravasi T, Suzuki H, Cannistraci CV, Katayama S, Bajic VB, et al (2010) An atlas of combinatorial transcriptional regulation in mouse and man. Cell 140: 744–752. 20211142 10.1016/j.cell.2010.01.044 PMC2836267

[20] Glossary of Terms. Machine Learning 30: 271–274.

[21] Consortium TU (2011) Ongoing and future developments at the Universal Protein Resource. Nucleic Acids Res 39: D214–D219. 21051339 10.1093/nar/gkq1020 PMC3013648

[22] Kawashima S, Ogata H, Kanehisa M (1999) AAindex: Amino Acid Index Database. Nucleic Acids Res 27: 368–369. 9847231 10.1093/nar/27.1.368 PMC148186

[23] Hall M, Frank E, Holmes G, Pfahringer B, Reutemann P, et al (2009) The WEKA data mining software: an update. SIGKDD Explor Newsl 11: 10–18.

[24] Tasan M, Tian W, Hill DP, Gibbons FD, Blake JA, et al (2008) An en masse phenotype and function prediction system for Mus musculus. Genome Biol 9 Suppl 1S8. 10.1186/gb-2008-9-s1-s8 PMC2447542 18613952

